# Supplementary material for: Induction of Extracellular Hydroxyl Radicals Production in the White-Rot Fungus Pleurotus eryngii for Dyes Degradation: An Advanced Bio-oxidation Process
Source: J Fungi (Basel). 2024 Jan 7;10(1):52. doi: 10.3390/jof10010052 (PMC10821177; doi:10.3390/jof10010052)
Supplement: Supplementary file 1 [file jof-10-00052-s001.zip › jof-2771238-supplementary.pdf]

Induction of Extracellular Hydroxyl Radicals Production in the White-Rot Fungus *Pleurotus eryngii* for Dyes Degradation: An Advanced Bio-oxidation Process

Table S1. Dyes used in the present study, showing their names and abbreviations, chemical structures, and absorption maxima in the visible spectrum, determined at pH 2.

Type	**Name** (abbreviation), λ_{max}	**Name** (abbreviation), λ_{max}	**Name** (abbreviation), λ_{max}
Anthraquinone	**Acid Black 48** (AB48), **657 nm** 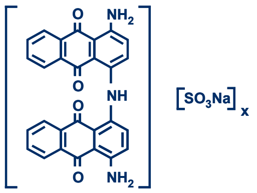	**Acid Blue 45** (AB45), **587 nm** 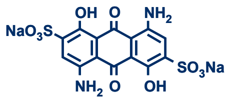	**Acid Green 25** (AG25), **608 nm** 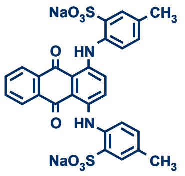
	**Reactive Blue 19** (RB19), **592 nm** 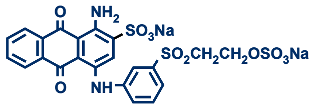		
Azo	**Acid Red 88** (AR88), **502 nm** 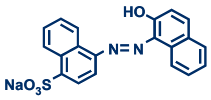	**Acid Yellow 17** (AY17), **402 nm** 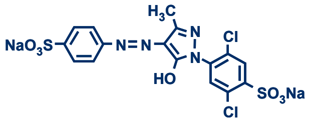	**Chromotrope 2R** (C2R), **505 nm** 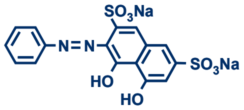
	**Crocein Orange G** (COG), **480 nm** 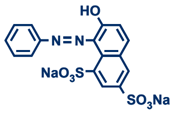	**Methyl Orange** (MO), **474 nm** 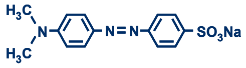	**New Coccine** (NC), **506 nm** 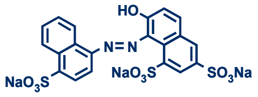
	**Orange II** (OII), **485 nm** 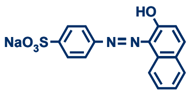	**Tartrazine** (TA), **427 nm** 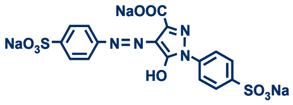	**Tropaeolin O** (TO), **388 nm** 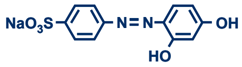

Table S1. Continuation.

Type	**Name** (abbreviation), λ_{max}	**Name** (abbreviation), λ_{max}	**Name** (abbreviation), λ_{max}
Diazo	**Acid Black 24** (AB24), **608 nm** 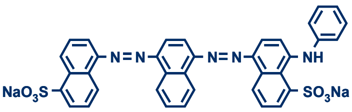	**Acid Blue 113** (AB113), **549 nm** 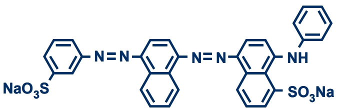	**Acid Orange 63** (AO63), **425 nm** 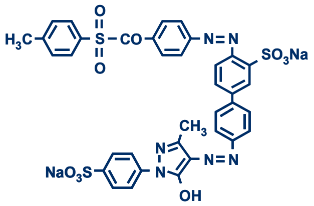
	**Ponceau SS** (PSS), **513 nm** 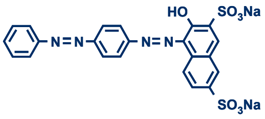	**Reactive Black 5** (RB5), **596 nm** 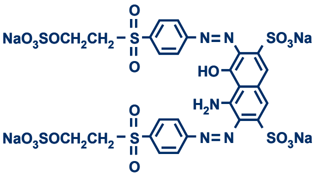	
Heterocycle	**Azure B** (AB), **645 nm** 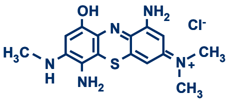	**Neutral Red** (NR), **530 nm** 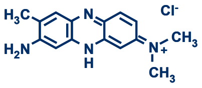	
Indigo Phthalocyanine	**Indigo Carmine** (IC), **609 nm** 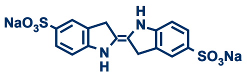	**Iron(III)phthalocyanine** (I3P), **631 nm** 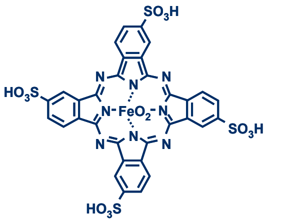	
Triarylmethane	**Brilliant Green** (BG), **624 nm** 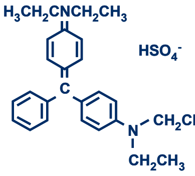	**Bromophenol Blue** (BB), **436 nm** 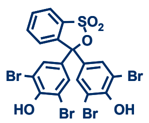	**Cresol Red** (CR), **434 nm** 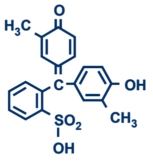
	**Crystal Violet** (CV), **597 nm** 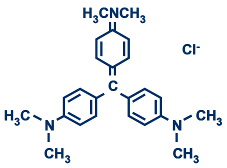	**Methyl Blue** (MB), **601 nm** 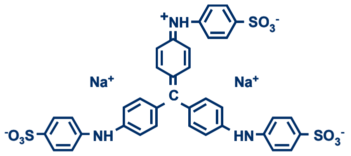	

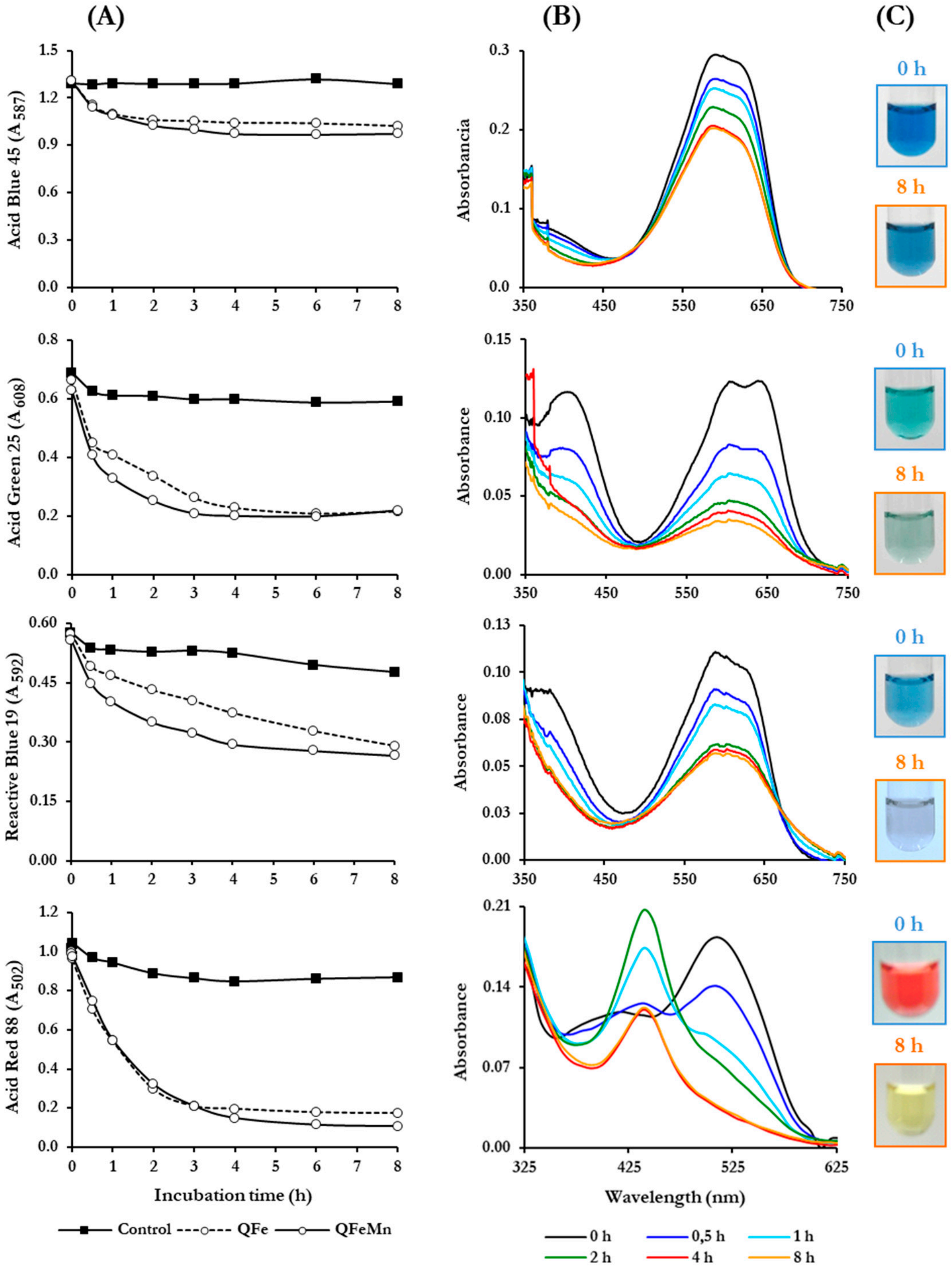

Figure S1. Advanced oxidation of dyes by *P. eryngii*. (A) Time course of decolourisation at λ_{max} of each dye; (B) changes in visible spectra of diluted samples from incubations with Mn^{2+} along the incubation time; (C) pictures of QFeMn incubation samples taken at 0 and 8 hours. Incubations contained 7-day-old washed mycelium (50 ± 5 mg, dry weight), 30 ml of 20 mM phosphate buffer, pH 5.0, dyes 50 µM, DBQ 500 µM, Fe^{3+} 100 µM-Oxalate 300 µM (incubation QFe) and Mn^{2+} 100 µM (incubation QFeMn). Control incubations lacked DBQ, iron complex and Mn^{2+}.

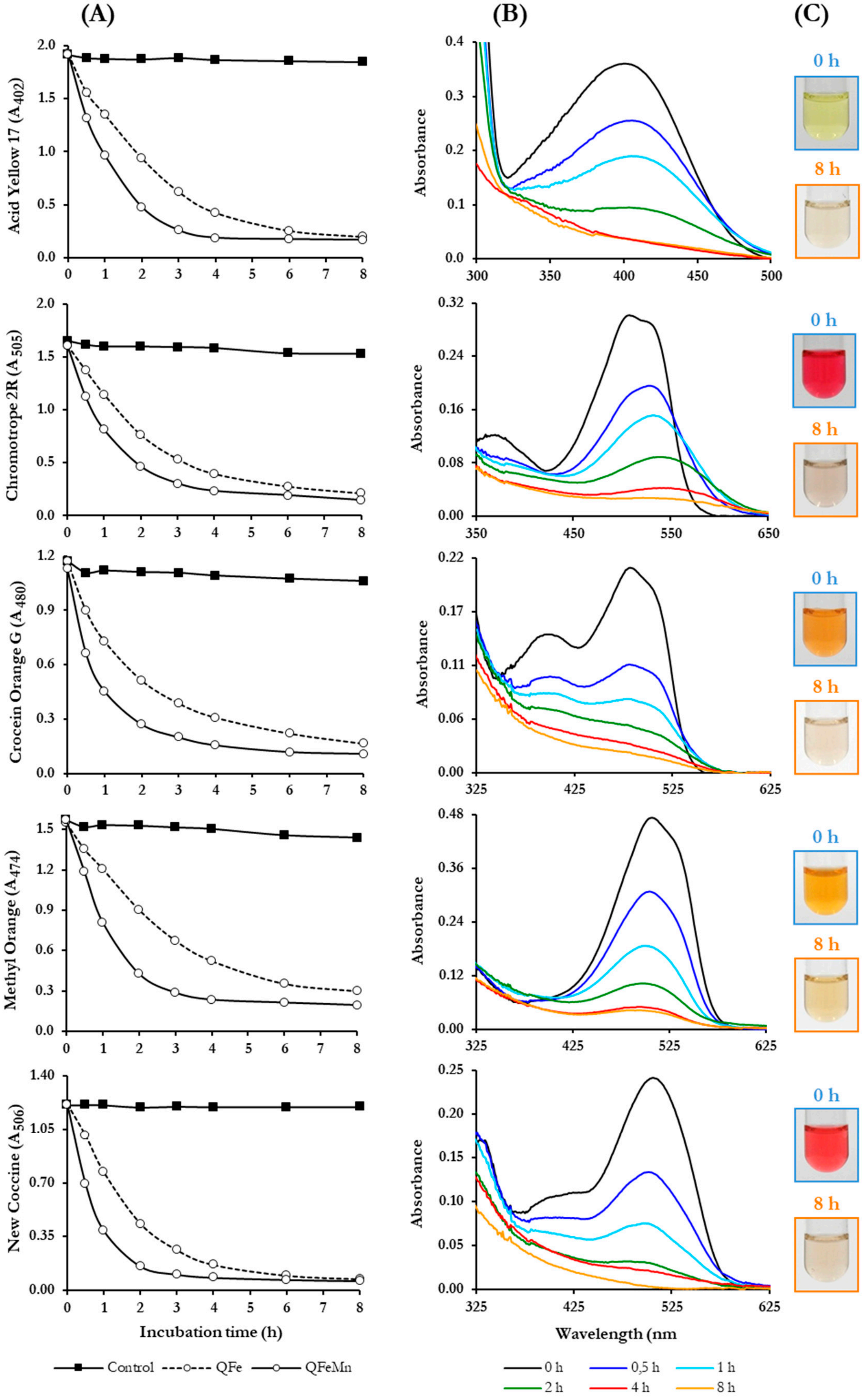

Figure S1. Continuation.

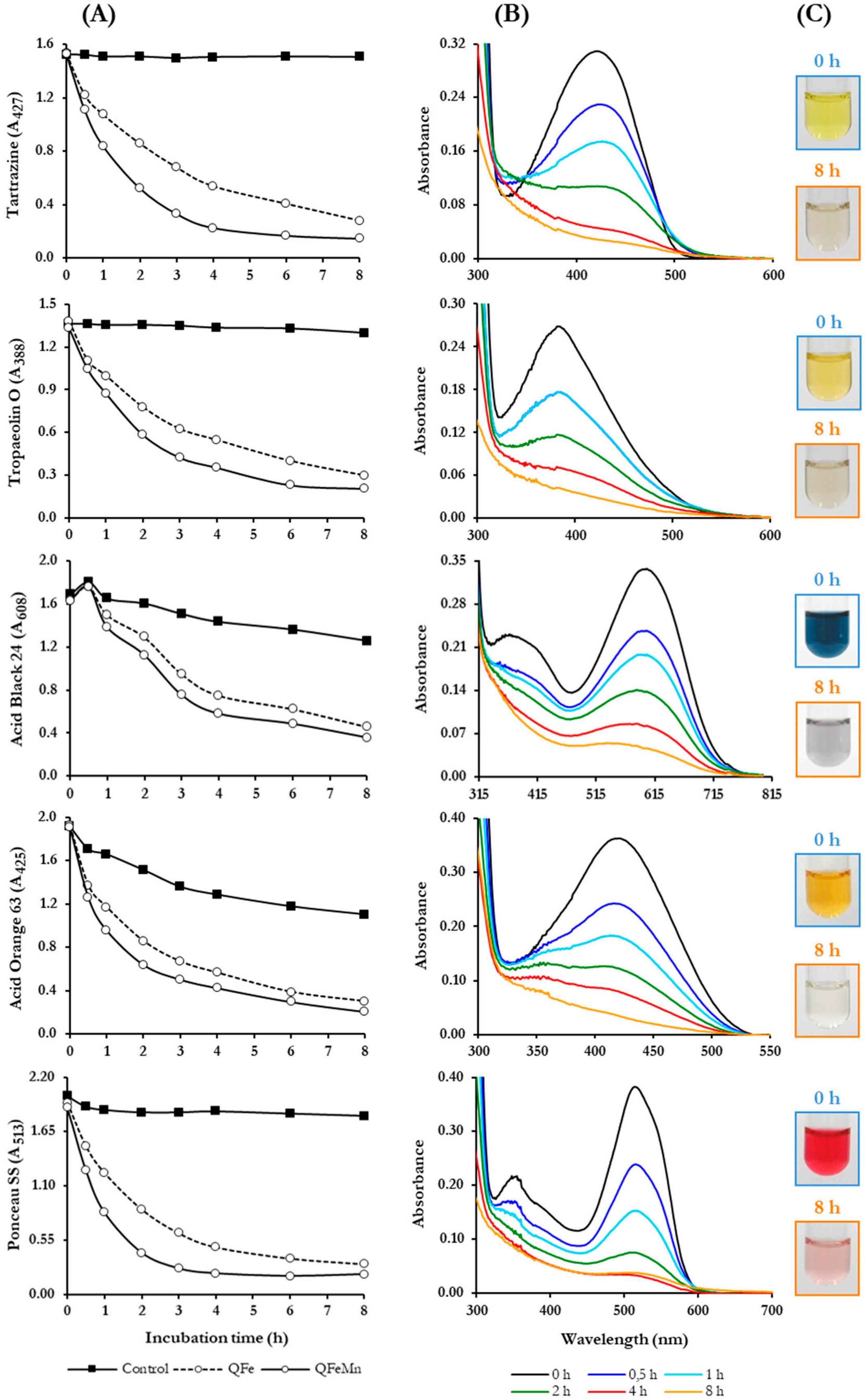

Figure S1. Continuation.

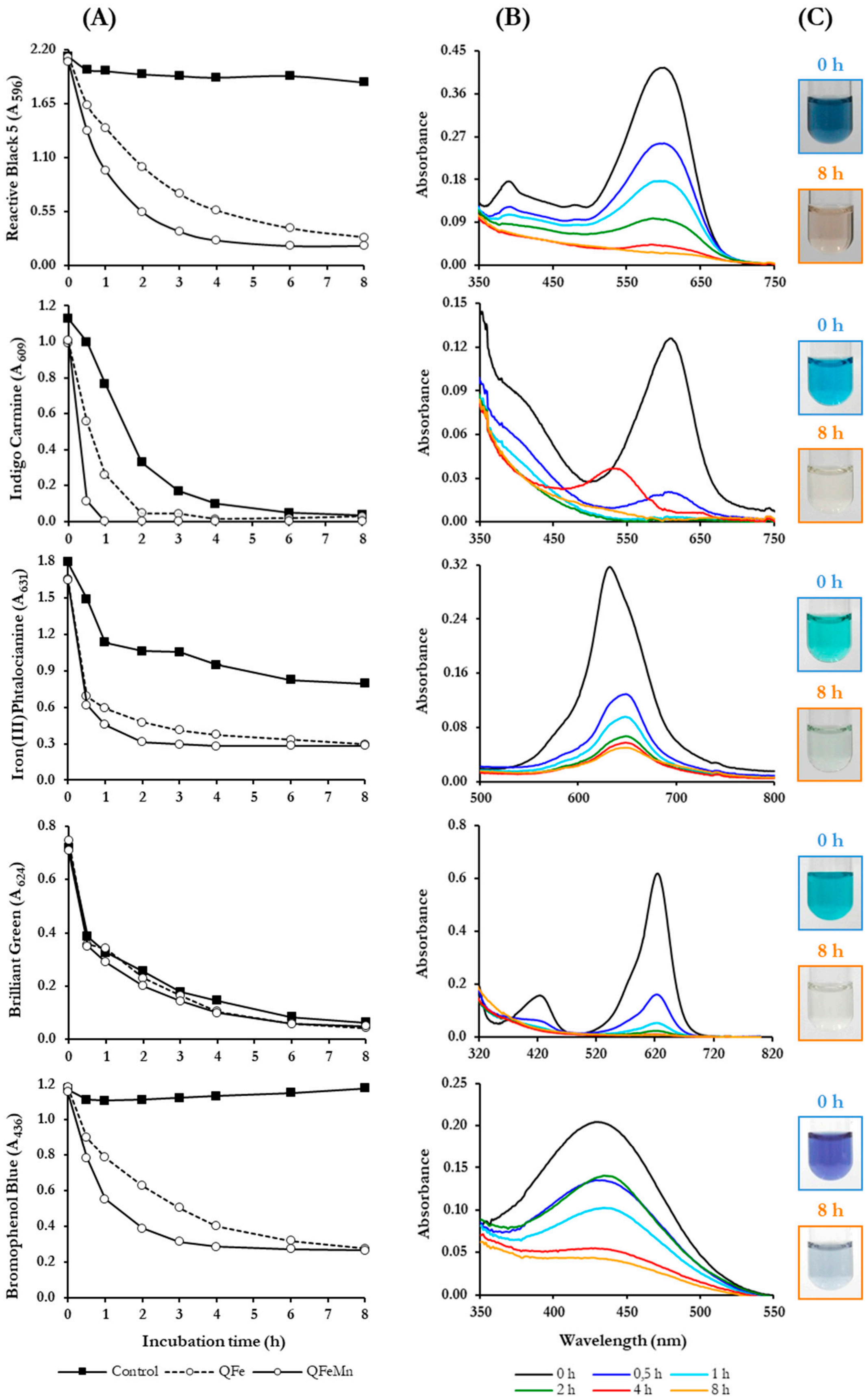

Figure S1. Continuation.

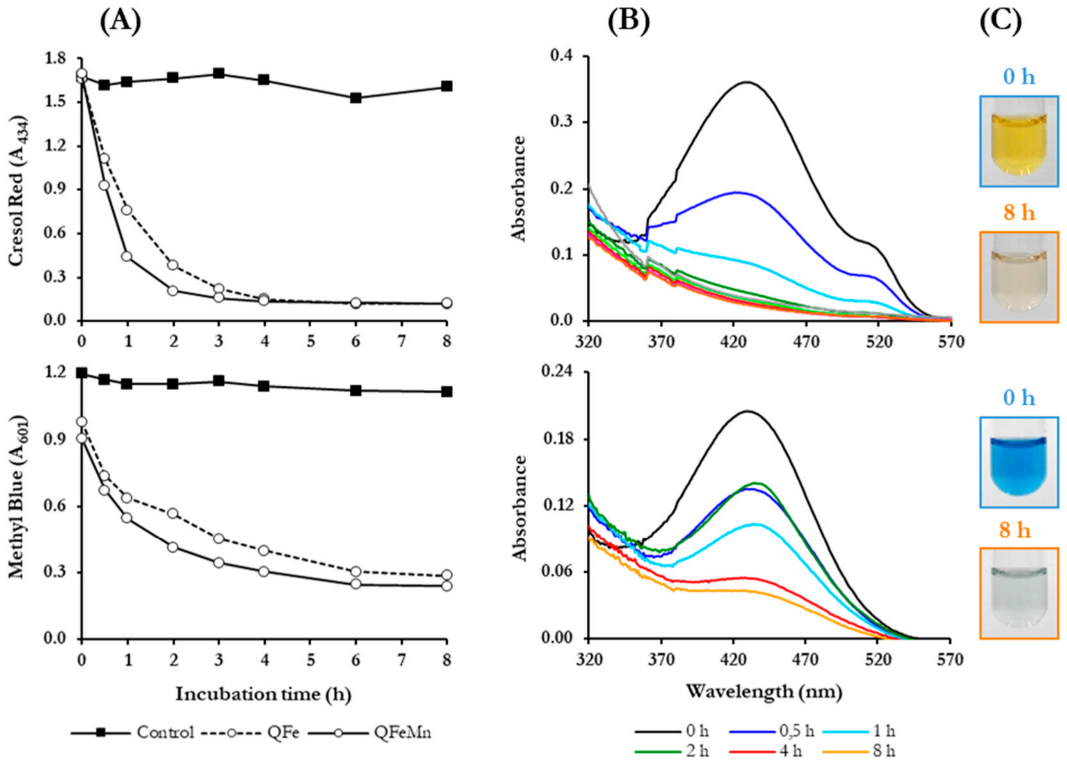

Figure S1. Continuation.

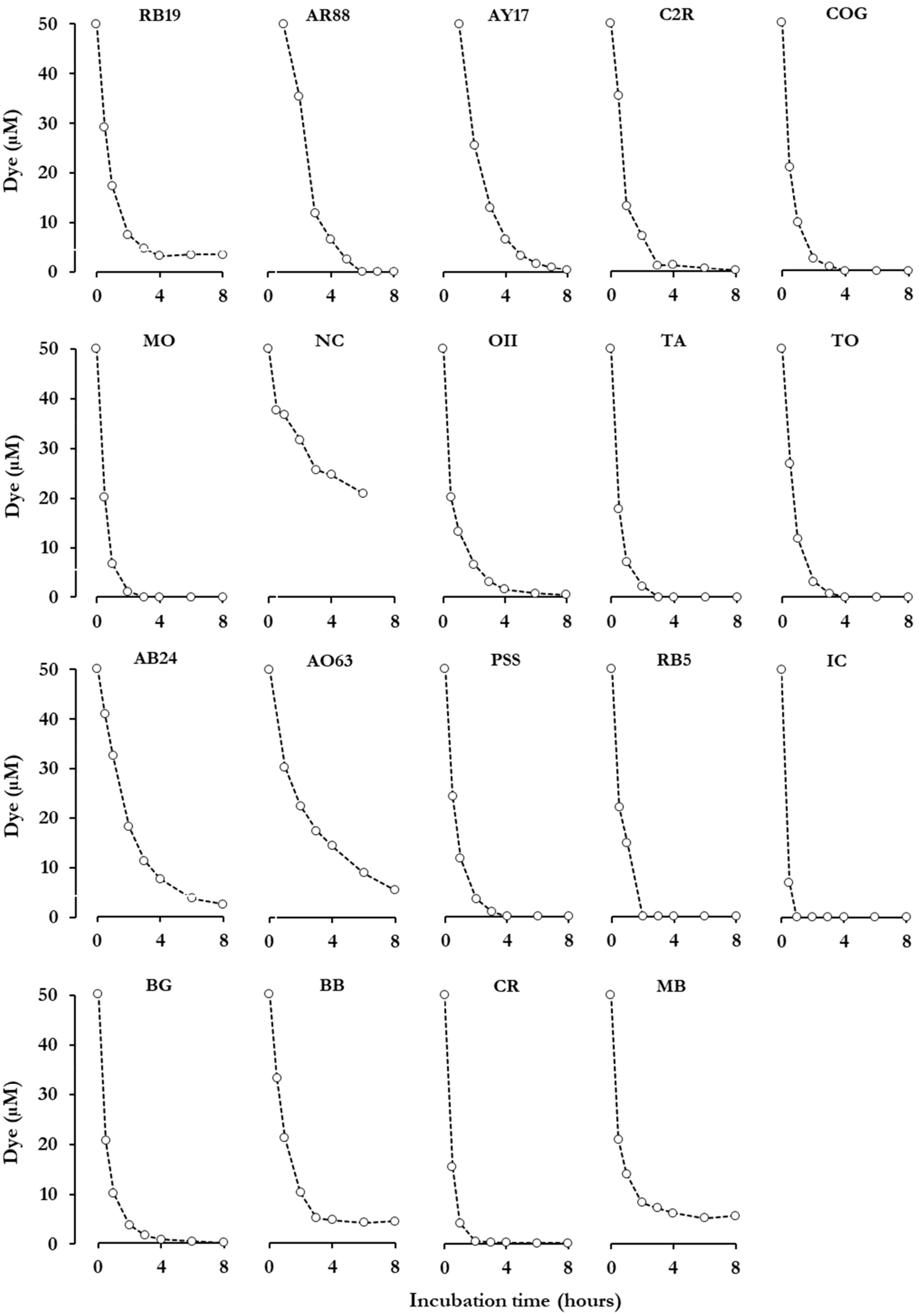

Figure S2. Advanced bioxidation of dyes by *P.eryngii*, analysed by HPLC. Incubations contained 7-day-old washed mycelium (50 ± 5 mg, dry weight), 30 ml of 20 mM phosphate buffer, pH 5.0, dyes 50 µM, DBQ 500 µM, Fe^{3+} 100 µM-Oxalate 300 µM and Mn^{2+} 100 µM (QFeMn incubation). Control incubations, which lacked DBQ, iron complex and Mn^{2+}, were analysed spectrophotometrycally and the results are shown in Figure S1.
